# Humanism and Psychosis: Working With Patients Who Have Potential Medical Comorbidities Along With Mental Illness

**DOI:** 10.7759/cureus.66903

**Published:** 2024-08-14

**Authors:** Mona Singh, Davin Agustines

**Affiliations:** 1 Osteopathic Medicine, Western University of Health Sciences, Pomona, USA; 2 Psychiatry, Olive View University of California Los Angeles Medical Center, Los Angeles, USA

**Keywords:** patient-centered care healthcare disparities, patients experiencing homelessness, medical mistrust, schizophrenia, marginalized

## Abstract

Marginalized groups, such as Black participants experiencing homelessness and diagnosed with schizophrenia, often face significant barriers to care. Improvements in treatment can be achieved by incorporating patient views, addressing stigmas, avoiding medical jargon, respecting patient preferences, and demonstrating transparency and positive affect. We discuss one example in our case report where a newly unhoused woman with schizophrenia, highlighting the impact of medical mistrust, discrimination, and ineffective communication in mental health care. We retrospectively used the Brief Psychiatric Rating Scale (BPRS) score to assess the severity of the patient's psychiatric condition after her workup. The severity of the BPRS scale is graded as mild (31-40 total score), moderate (41-52 total score), and severe (above 52 total score). Using this scale and our case report, we aim to highlight the importance of emphasizing the rationale of the plan of care to patients, explaining their diagnoses, and reasoning of diagnostics without using medical jargon.

## Introduction

Perceived racism is a concept that is associated with cultural mistrust in Black participants, especially with respect to the field of psychiatry. It has been found that perceived racism predicts cultural mistrust and non-clinical paranoia in Black participants, and some paranoia may also be viewed as a healthy and adaptive strategy for this population [1]. Unfortunately, healthy suspiciousness may be conflated with delusion by mental health clinicians [2]. When engaging all patients, including the Black population, it can be more fruitful to educate patients about the rationale for various diagnostic tests rather than overly emphasizing their mental illness to minimize this type of misunderstanding. Investing time in building rapport, ensuring effective communication, and understanding patients' specific needs can expedite treatment adherence and cooperation, ultimately leading to improved healthcare quality.

To illustrate combating cultural mistrust experienced by Black participants with a psychiatric disorder, we discuss a case report of a female patient experiencing homelessness who self-reported being Black. Her presentation in the psychiatric emergency department revealed symptoms of psychosis, including delusions and hallucinations, compounded by a history of mistrust of medical professionals. Initially, there was uncertainty regarding the patient's pregnancy status. However, due to a lack of a clear explanation for the necessary labs and ultrasound, she refused treatment and was labeled as hostile and uncooperative. Her severe psychiatric symptoms were overshadowed, leading to a quick diagnosis of psychosis with an exaggeration of her delusions and hallucinations. This highlights the urgent need for patient-centered care and transparent communication to foster trust and cooperation, especially among minorities with long-standing medical mistrust.

In our case report, we utilized the Brief Psychiatric Rating Scale (BPRS), a commonly used scale to establish the possible presence and severity of a variety of psychiatric disorders [3]. It is one of the oldest rating scales, used by clinicians and researchers to evaluate several psychiatric symptoms such as depression, anxiety, and psychosis. The 24 items are graded on a scale from one (not present) to seven (extremely severe), and 0 is marked if any item is not assessed. The total scores range between 24 and 168, with higher scores indicating more severe psychopathology [4]. The severity of the BPRS scale is graded as mild (31-40 total score), moderate (41-52 total score), and severe (above 52 total score) [5]. We utilized a retrospective BPRS scale in our case, determining the score after the patient's medical workup. Using this scale and our case report, we aim to highlight the importance of emphasizing the rationale of the plan of care to patients, explaining their diagnoses, and reasoning of diagnostics without using medical jargon.

## Case presentation

The patient, a 43-year-old Black woman experiencing homelessness, was admitted to the emergency department (ED) after being found walking in the street while holding a butter knife. When approached by officers, she refused to give them the knife, resulting in the police bringing her to the ED. She endorsed auditory hallucinations of "dogs whining" and visual hallucinations of distorted faces. Her urine beta-human chorionic gonadotropin (B-Hcg) also came back with a positive result, but she flatly refused to allow any further laboratory examination to verify. Her refusal of diagnostic examinations was labeled as evidence of hostility and delusions by the ED staff, and she was transferred to an inpatient psychiatric ward for further treatment. 

Upon transfer to the ward, the patient was guarded, refusing all diagnostic tests and labs, saying, “I’m not pregnant; my stomach is flat.” She was started on olanzapine, which was uptitrated to 7.5 mg daily, to target the positive symptoms of psychosis that she endorsed. While in the inpatient psychiatric unit, the treating psychiatrist and medical student spent 20-30 minutes daily with the patient establishing rapport and thoroughly explaining why she needed labs to monitor electrolytes and needed a serum B-hcg and first trimester ultrasound to verify if she was pregnant. Taking the time to build trust and explaining the rationale behind her medical management eventually allowed the patient to comply. The provider and medical student reassured the patient and coordinated with her by offering to be beside her during the blood draw and ensuring she had a meal and fluids. She ultimately agreed to all further tests and treatment. Her BPRS score at this point had gone from severe at 61 to moderate at 51. 

After initially agreeing to allow the ultrasound, she then refused after encountering the radiology technician and would only allow abdominal ultrasound and not a transvaginal ultrasound. She became increasingly guarded and mistrustful of the treatment team, believing that she had been tricked into agreeing to an invasive procedure. Her positive symptoms of psychosis also worsened at this time. She endorsed having increased visual hallucinations of women sitting beside her bed and auditory hallucinations of sounds of “whining” and “crying” in the middle of the night. She did agree to allow further lab draws and was determined to be not pregnant through B-hcg values. The pregnancy's first-trimester ultrasound showed a transabdominal scanning bladder inadequately filled to visualize the uterus. The patient’s B-hcg was 2.9 mIU/mL and the repeat was 1.5 mIU/mL, concluding a negative pregnancy. This is the second instance of the patient's unfamiliarity with medical procedures and not understanding why she needed specific tests which quickly developed into hostility. It also exacerbated her symptoms of psychosis. 

The treating psychiatrist and medical student decreased the patient's unhealthy suspicion that they were going to do something harmful toward her by instead normalizing her concerns as a human and creating an environment of understanding to meet the patient’s healthcare needs. The patient thereafter remained stable on her medication regimen, was without any behavioral or aggressive issues, and became less paranoid and withdrawn. Treatment with olanzapine helped reduce her positive symptoms of psychosis secondary to her diagnosis of schizophrenia. Ultimately, the patient was discharged to a shelter after a 28-day hospitalization. The patient's BPRS score upon discharge was mild at 38.

## Discussion

The patient initially presented as extremely guarded and paranoid upon arrival at the psychiatric ED. In addition to her auditory and visual hallucinations, her suspicion and paranoia were worsened by the medical team’s initial failure to effectively communicate the rationale behind the diagnostic tests requested of her. Despite her healthy suspicion, her concerns were overlooked, and she was instead hastily labeled as delusional and paranoid. This underscores the perpetuation of institutionalized discrimination and stigma against certain racial groups, individuals experiencing homelessness, and those with mental illnesses, particularly among Black participants with conditions like schizophrenia. Through patience and empathy and in combination with antipsychotic medication to target her psychosis, medical testing was successful after treating her as a human and not just a patient to treat.

Patients with multiple healthcare disparities, including socioeconomic status, gender, racial and ethnic backgrounds, and serious mental illness, can collectively exacerbate medical mistrust and discrimination within the healthcare system, particularly evident in inpatient psychiatric settings. Establishing trust between physicians and patients, forming strong connections, and empathizing with patients is therefore imperative. In this patient's case, the care team spent time building rapport and explaining the necessity of labs and diagnostic tests to confirm her pregnancy and tailor medication accordingly, particularly concerning atypical antipsychotics like risperidone, which pose an increased risk of birth defects [6]. Within the first four days of her inpatient psychiatric stay, after thorough communication with the patient, she became compliant and cooperative with all tests and treatments. This underscores the importance of human connection and avoiding the excessive use of medical jargon to ensure patient understanding and agreement with further workup.

Establishing trust with patients, including those from marginalized populations like Black participants experiencing homelessness and psychiatric illnesses, is crucial for high-quality care. Research shows that one barrier to medical care for physical illnesses of patients with psychiatric conditions is the belief by physicians that their patients' verbal complaints stem from their mental illness [7]. Psychological factors like cultural mistrust that are associated with attitudes toward healthcare providers in the larger Black population may also be found when they have severe mental illness [7]. The patient had complaints of dizziness and being afraid of a syncope episode after drawing blood but was disregarded multiple times by staff as they presumed it was a part of her psychosis. Once the patient informed the treating psychiatrist and medical student, her complaint was quickly heard as we provided her with an extra meal so that she would have an adequate food supply in case a syncope episode took place. In general, cultural mistrust is experienced by Black participants for physical health and mental health. This shows the clinicians' own biases may be influential in the treatment of Black participants with psychiatric conditions. Being cognizant of biases and not making assumptions about patients' subjective experiences are therefore important to promote high-quality healthcare. 

Moreover, interdisciplinary communication is essential. Addressing the patient's pregnancy through effective communication could have potentially prevented her admission to the psychiatric unit for medical management and streamlined her care for psychosis. Collaborating with patients to address medical issues and enhancing medical trust through rapport-building would afford more opportunities for effective medical workups. Studies have also confirmed how an openness to cultural diversity is critical in both clinical practice and approaches toward future research [8]. Research has shown one way to reduce inequalities is through culturally informed approaches both within existing clinical practice and by moving towards a more diverse and representative workforce at all levels [8]. By establishing trust with the patient, understanding her needs without stigmatization, allowing her a safe space to be authentically herself, and engaging her in her treatment, we were able to mitigate both her positive and negative symptoms of psychosis secondary to schizophrenia. Throughout her hospitalization, efforts were made to build rapport and elucidate the rationale behind medical interventions, such as the B-Hcg lab and first-trimester ultrasound, to determine pregnancy status. Within a short period, the patient demonstrated improved adherence and cooperation, reflected in a decrease in her BPRS score from 61 to 38. 

## Conclusions

This patient's case emphasizes the need for patient-centered approaches in healthcare delivery, particularly for marginalized populations such as Black participants experiencing homelessness. To help mitigate healthcare disparities, it is imperative to approach each patient on an individual level, even if they present challenges. Rather than stigmatizing or overdiagnosing patients, fostering a sense of normalcy can facilitate better healthcare outcomes by promoting treatment adherence and cooperation. By addressing the unique needs of individuals with various healthcare disparities, we can work towards achieving holistic improvements in health outcomes across the population. It is essential to recognize and address the intersectionality of factors contributing to healthcare disparities to promote equitable and effective healthcare delivery for all.

## References

[1] Faber SC, Khanna Roy A, Michaels TI, Williams MT (2023). The weaponization of medicine: early psychosis in the Black community and the need for racially informed mental healthcare. Front Psychiatry.

[2] Mosley DV, Owen KH, Rostosky SS, Reese RJ (2017). Contextualizing behaviors associated with paranoia: perspectives of Black men. Psyc Men Masculinity.

[3] Zanello A, Berthoud L, Ventura J, Merlo MC (2013). The Brief Psychiatric Rating Scale (version 4.0) factorial structure and its sensitivity in the treatment of outpatients with unipolar depression. Psychiatry Res.

[4] Hofmann AB, Schmid HM, Jabat M (2022). Utility and validity of the Brief Psychiatric Rating Scale (BPRS) as a transdiagnostic scale. Psychiatry Res.

[5] Leucht S, Kane JM, Kissling W, Hamann J, Etschel E, Engel R (2005). Clinical implications of Brief Psychiatric Rating Scale scores. Br J Psychiatry.

[6] Singh KP, Singh MK, Singh M (2016). Effects of prenatal exposure to antipsychotic risperidone on developmental neurotoxicity, apoptotic neurodegeneration and neurobehavioral sequelae in rat offspring. Int J Dev Neurosci.

[7] Whaley AL (2010). Psychiatric and psychological predictors of self-reported health of African Americans with severe mental illness. Psychiatr Serv.

[8] Hui A, Rennick-Egglestone S, Franklin D (2021). Institutional injustice: Implications for system transformation emerging from the mental health recovery narratives of people experiencing marginalisation. PLoS One.

